# Ribosome reactivates transcription by physically pushing RNA polymerase out of transcription arrest

**DOI:** 10.1073/pnas.1919985117

**Published:** 2020-04-01

**Authors:** Flint Stevenson-Jones, Jason Woodgate, Daniel Castro-Roa, Nikolay Zenkin

**Affiliations:** ^a^Centre for Bacterial Cell Biology, Biosciences Institute, Newcastle University, NE2 4AX Newcastle Upon Tyne, United Kingdom

**Keywords:** transcription–translation coupling, RNA polymerase, ribosome, backtracking, conflicts with replication

## Abstract

In bacteria, the first two steps of gene expression—transcription and translation—are spatially and temporally coupled. Uncoupling may lead to the arrest of transcription through RNA polymerase backtracking, which interferes with replication forks, leading to DNA double-stranded breaks and genomic instability. How transcription–translation coupling mitigates these conflicts is unknown. Here we show that, unlike replication, translation is not inhibited by arrested transcription elongation complexes. Instead, the translating ribosome actively pushes RNA polymerase out of the backtracked state, thereby reactivating transcription. We show that the distance between the two machineries upon their contact on mRNA is smaller than previously thought, suggesting intimate interactions between them. However, this does not lead to the formation of a stable functional complex between the enzymes, as was once proposed. Our results reveal an active, energy-driven mechanism that reactivates backtracked elongation complexes and thus helps suppress their interference with replication.

## Significance

Transcription and translation are coupled in bacteria, meaning that translation of the nascent mRNA by ribosome happens simultaneously with its synthesis by RNA polymerase (RNAP). Uncoupling of transcription from translation may lead to arrest of transcription via backtracking of RNAP along the template, leading to conflicts with DNA replication, although the molecular mechanisms involved are unclear. We assembled an in vitro coupled transcription–translation system in which both machineries can be controlled with single-nucleotide precision. We showed that through a direct interaction, the coupled ribosome, physically pushes RNAP from the backtracked state, thereby reactivating transcription. This rescue ability of the ribosome prevents inhibition of translation by transcriptional backtracking and mitigates conflicts of backtracked RNAPs with replication at protein-coding genes.

Unlike in eukaryotes, where transcription and translation are separated by a nuclear envelope, in bacteria these processes are coupled. This coupling not only is important for synchronization of the processes and regulation of gene expression (1–3), but also has been linked to genomic stability (4) by mitigating the backtracking of transcription elongation complexes (ECs). Backtracking is a phenomenon in which the EC slides backward along the DNA template and the nascent mRNA, resulting in loss of the 3′ end of the mRNA from the active center and thereby inactivating RNA synthesis by the EC, which remains stably bound to DNA. If not reactivated, backtracked ECs form strong roadblocks on DNA that can cause transcriptional traffic jams (5) and collisions with replication machinery, leading to double-strand DNA breaks (4).

The elongation factors GreA and GreB (in *Escherichia coli*; many bacteria have only one Gre factor) can reactivate backtracked ECs by promoting a transcript cleavage reaction at the active site that generates a new 3′ end (6). However, these factors are not essential, suggesting that coupling could play a critical role in mitigating transcription backtracking. In contrast to RNAP, the ribosome can move backward along mRNA by only one codon into the pretranslocated state (7) and thus blocks long backtracking of the EC if both machineries are sufficiently close to each other on mRNA (3). However, whether the leading ribosome can actively reverse EC backtracking, or if the backtracked EC forms a roadblock on mRNA and inhibits ribosome translocation and thus translation, is unknown.

## Results

### Coupled Ribosome Pushes Backtracked ECs Forward.

**Fig. 1.**
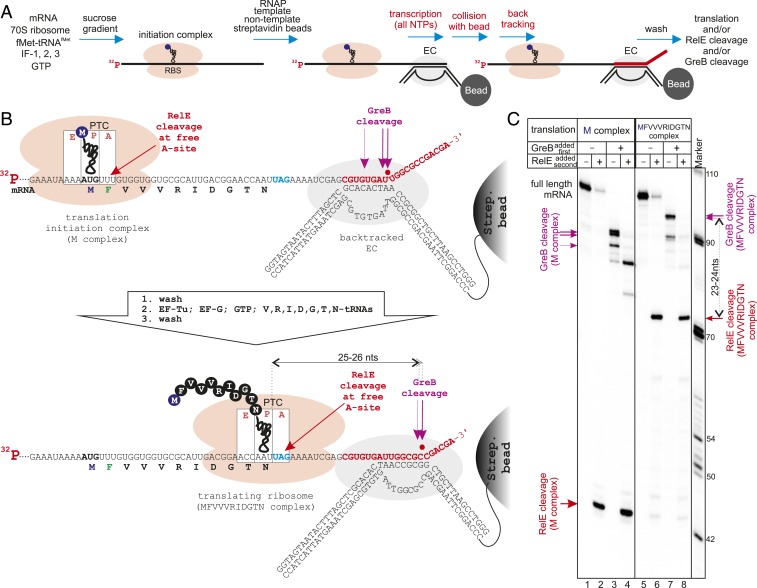
Ribosomes push backtracked RNAP. (*A*) Initial steps of assembly of the coupled transcription–translation system. Purified translation initiation complexes (M complexes) are coupled to an assembled transcription EC and immobilized on streptavidin beads, and the EC is allowed to transcribe to the position that causes stable backtracking. (*B* and *C*) Scheme and results of the experiment. EC coupled to translation M complex is walked to a position that causes stable backtracking (because of collision with a streptavidin bead). Then ribosome translates an 11-aa peptide approaching the backtracked EC before it stalls with a UAG stop codon in the A-site (lanes 5 to 8). The positions of RNAP and the ribosome are determined by GreB and RelE cleavage on 5′ radiolabeled mRNA, respectively.

To investigate the consequences of the encounters of backtracked ECs with a coupled translating ribosome, we developed an in vitro coupled transcription–translation system in which both machineries can be translocated in a controlled stepwise manner and monitored with single nucleotide precision (Fig. 1 *A* and *B*). The translation initiation complex (M complex with fMet-tRNA^fMet^ in the ribosome P-site) was formed on a 5′-radiolabeled mRNA (mRNA25). The purified M complex was then coupled to transcription by using its mRNA as a transcript in the assembly of a transcription EC with RNAP and complementary template and nontemplate DNA strands. The coupled system was immobilized on streptavidin beads via a biotin tag on the 3′ end of the nontemplate DNA strand, thereby ensuring the presence of only fully assembled ECs and allowing exchange of soluble components by washing of the beads.

After addition of all nucleotides, the EC transcribed but stopped before reaching the end of the DNA (red nucleotides in Fig. 1 *A* and *B*; also see *SI Appendix*, Fig. S1*A*), because of the latter’s attachment to the solid phase (streptavidin bead), as described previously (8). Such a stop induced stable backtracking of this EC by 12, 13 and 16 bp, as revealed by rapid (5 s) cleavage by GreB, which cleaves the backtracked transcript in the RNAP active center, thus marking its precise position (Fig. 1 *B* and *C* lane 3). The position of the ribosome on the mRNA and the efficiency of coupling can be judged by cleavage with RelE toxin, which cleaves mRNA specifically between the second and third nucleotides of the codon in the vacant A-site of the posttranslocated ribosome (9) (Fig. 1*B*). As shown in Fig. 1*C* (lanes 2 and 4), most of the immobilized ECs contained translation M complexes on their transcripts (before and after GreB cleavage).

Next, the ribosome was allowed to translate toward the backtracked EC by the addition of individual aminoacylated tRNAs (aa-tRNAs, in the form of ternary complexes with EF-Tu/GTP), translocation factor EF-G, and GTP. Because RelE cleavage is most efficient when the A-site contains a UAG stop codon (9), we placed UAG after the 11-aa open reading frame (ORF) (Fig. 1*B*). As can be judged from the RelE cleavage pattern shown in Fig. 1*C* (lane 6), most of the ribosomes translated 11 codons and stalled with UAG in the A-site. Interestingly, this resulted in the forward translocation of backtracked ECs by 6 to 10 bp (Fig. 1*C*, compare lanes 3 and 7), converting them from the initial 12- to 16-bp backtracked states into 6- to 7-bp backtracked states. All the forward translocated ECs remained coupled to translation, as revealed by RelE cleavage (Fig. 1*C*, lanes 6 and 8). Transcription elongation factors NusA and NusG can be associated with transcribing EC in the cell (with NusG being implicated in coupling of transcription and translation) (10) and thus may affect the observed reversal of backtracking; however, the addition of NusA and NusG factors before translation had no effect on the reversal of backtracking or on the extent of the EC translocation (*SI Appendix*, Fig. S2). These results indicate that the backtracked EC does not inhibit coupled translation of the nascent mRNA, but instead, the translating ribosome can actively reverse backtracking.

### Minimal Distance between Interacting RNAP and Ribosome.

**Fig. 2.**
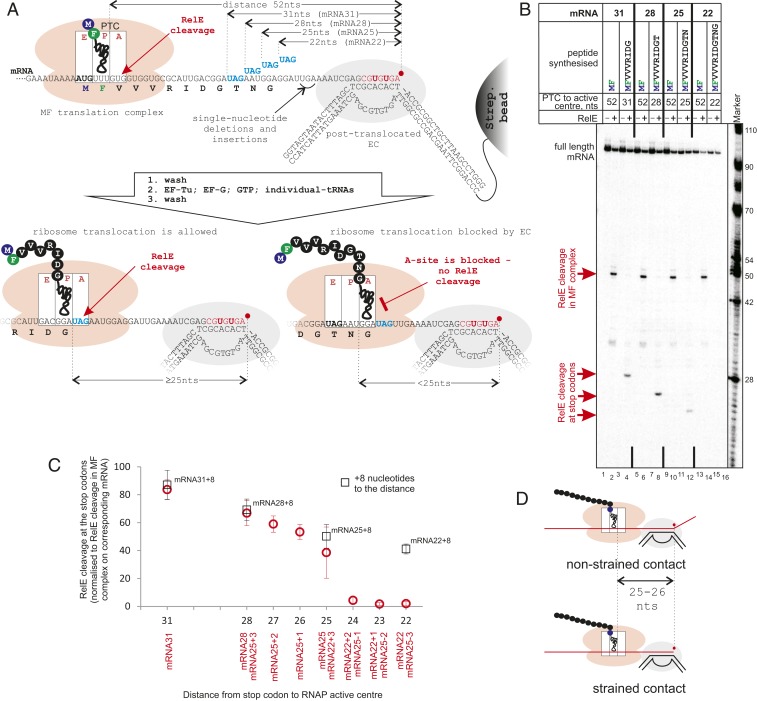
The distance between the coupled ribosome and RNAP interacting on mRNA. (*A* and *B*) Scheme and results of the experiment. MF complexes are coupled to ECs as in Fig. 1*A*, except here the EC is walked to the abasic position, where it is stabilized in the posttranslocated state (*SI Appendix*, Fig. S3*A*) and cannot extend mRNA further. mRNAs contain UAG stop codons (to standardize efficiency by RelE cleavage among mRNAs) at different distances from the RNAP active center. Ribosomes translate 9-, 10-, 11-, or 12-aa peptides toward the EC before they reach UAG stop codons. The availability of UAG in the A-site, as a measure of successful translocation of the ribosome, is tested by RelE cleavage (lanes 4, 8, 12, and 16). Ribosomes that came in contact with RNAP and cannot translocate have peptidyl-tRNA in the A-site, making them inaccessible for RelE (lane 16). (*C*) Efficiencies of RelE cleavage at UAGs located at various distances from the RNAP active center. The positions of one, two, and three nucleotide deletions and insertions in mRNAs are shown in *A* and in *SI Appendix*, Fig. S1*A*. Data points are means and error bars are SDs from 3 to 13 experiments. Black squares are the RelE cleavage efficiencies on the corresponding mRNAs when the EC was walked 8 bp (on a template without abasic site) further away from stop codons. (*D*) The distances on mRNA between the RNAP active center and the ribosome’s PTC in both the “strained” contact, when the ribosome is forced into a stalled EC (*Bottom*), and the “nonstrained” contact, when ribosome translocation pushes the backtracked RNAP (*Top*) are the same, 25 to 26 nucleotides.

The distance between GreB and RelE cleavage sites on the mRNA after the ribosome reversed backtracking of the EC was 23 to 24 nucleotides (Fig. 1*C*, lanes 7 and 8), indicating that the distance between the peptidyl-transferase center (PTC) of the ribosome and the active center of RNAP on the mRNA was 25 to 26 nucleotides. To determine whether this distance reflects direct contact between the machineries and thus active pushing of the EC by the ribosome, we needed to measure the contact (minimal) distance on the mRNA between the translating ribosome and the transcribing RNAP. To do so, we stalled the EC in the posttranslocated state (instead of backtracked state). We argued that in the absence of a contact with the EC, the ribosome will have a vacant A-site in which RelE can bind and cleave mRNA. In contrast, on contact with the stalled posttranslocated EC (which cannot move forward), translocation of the ribosome would be inhibited, and the A-site would remain occupied by the peptidyl-tRNA, thus preventing RelE binding and cleavage (Fig. 2*A*). We used four different mRNAs containing UAG stop codons (to standardize RelE cleavage efficiency between mRNAs) located at various distances from the 3′ end of mRNA, that is, the active center of the posttranslocated RNAP (Fig. 2*A* and *SI Appendix*, Fig. S1*A*). A similar approach was previously used in vivo to measure the proximity of the ribosome to a termination hairpin required to disrupt the hairpin, with the expression level of the gene downstream of the hairpin reporting on the interaction (11).

The coupled systems on these mRNAs were prepared as shown in Fig. 1*A*, except that the translation elongation MF complexes (having MetPhe peptidyl-tRNA in the P-site; Fig. 2 *A* and *B*, lanes 2, 6, 10, and 14) were used as the starting point in place of M complexes. The coupled EC was transcribed (red nucleotides in Fig. 2*A*) to an abasic site in the template DNA (Fig. 2*A* and *SI Appendix*, Fig. S1*A*), which stabilized the EC in the posttranslocated state (as revealed by the resistance to pyrophosphorolysis, intrinsic and GreA cleavages, and very slow GreB cleavage; *SI Appendix*, Fig. S3*A*). However, the EC stalled at the abasic site was still able to incorporate an extra nucleotide during walking (*SI Appendix*, Fig. S3*A*, lane 1), and extend mRNA in the presence of the high levels of GTP required for translation (*SI Appendix*, Fig. S3*A*, lanes 10 and 11), which would affect measurement of the precise distance between the ribosome and RNAP. Therefore, we used 3′-dAMP as the last incorporated nucleotide before the abasic site, which fully abolished unwanted mRNA extension (*SI Appendix*, Fig. S3*A*, lanes 12, 21, and 22). The presence of 3′-dAMP at the 3′ end of mRNA did not change the translocation state of the EC (*SI Appendix*, Fig. S3*A*).

Note that in this experiment, the mRNAs were radiolabeled during transcription via incorporation of radioactive nucleotides into the mRNAs (bold red letters in Fig. 2*A*), and thus the RelE cleavage products become shorter with the ribosome approaching the EC. RelE cleavage revealed that ribosomes translating from the MF complexes reached the UAG stop codons when ribosome and RNAP active centers were 31, 28, and 25 nucleotides apart on the mRNA (mRNA31, mRNA28, and mRNA25, respectively) (Fig. 2*B*, lanes 4, 8, and 12 and Fig. 2*C*). However, no RelE cleavage at the UAG stop codon was observed on mRNA22 (Fig. 2*B*, lane 16 and Fig. 2*C*), indicating that 22 nucleotides between the ribosome’s PTC and RNAP active center is insufficient to allow the ribosome to translocate to the UAG stop codon and vacate the A-site for RelE. The absence of RelE cleavage at this stop codon was strictly dependent on the distance between the EC and the ribosome, as when the EC was walked further downstream by 8 bp (on a template without an abasic site; Fig. 2*C*, black open squares; *SI Appendix*, Fig. S1*A*) or translation was uncoupled (*SI Appendix*, Fig. S3*B*), cleavage at this stop codon was restored. The effects of one, two, and three nucleotide deletions and insertions in mRNA22 and mRNA25 (Fig. 2*C* and *SI Appendix*, Fig. S1*A*) revealed that 25 nucleotides between the ribosome’s PTC and RNAP active center was the minimal distance before translocation of the ribosome was physically blocked by the EC.

### Cooperation Rather than Functional Complex between the Coupled EC and Ribosome.

We observed the same distance of 25 to 26 nucleotides between ribosome and RNAP active centers during both “strained” (when the ribosome is forced into a stalled EC) and “nonstrained” (when ribosome translocation pushes the backtracked RNAP) contacts between them (scheme in Fig. 2*D*). This indicates that recovery of the EC from the backtracked state takes place through the physical contact between the ribosome and RNAP. The similarity of distances also suggests a common interface between the ribosome and RNAP during their contact on mRNA. However, whether this contact represents a complex of functionally and structurally interacting machineries is unclear. We argued that if the ribosome and EC that are in contact distance on mRNA form a stable ribosome/EC complex, stalling of the ribosome is likely to affect transcription of the EC. To test this, we analyzed transcription elongation by GreB-reactivated backtracked EC that was or was not within the contact distance with the translating ribosome (complexes in Fig. 1*B*). As shown in *SI Appendix*, Fig. S4, the kinetics of RNA extension was similar in both cases, suggesting that the contact with the stalled ribosome on mRNA does not hold back the EC. Therefore, this result suggests that there may be no tight complex formed between transcribing RNAP and translating ribosome. Whether or not NusG that interacts with both ribosome and RNAP (10) may facilitate formation of such a stable supercomplex is currently under investigation in a separate study.

## Discussion

Both RNAP and the ribosome move along their respective templates using a molecular ratchet powered by Brownian motion (12, 13). The translation machinery is further assisted by the elongation factor EF-G, which promotes translocation using the energy of GTP hydrolysis. This means that even if the posttranslocated ribosome shifts backward into the pretranslocated state (“translational backtracking”) (7), EF-G quickly reverts it into the posttranslocated state, thereby vacating the A-site for the next aa-tRNA. RNAP lacks similar energetic assistance, and inactive backtracked ECs remain stably stuck at the most thermodynamically favorable sequence of nucleic acids. Our results show that in addition to passive prevention of backtracking (3), translocation of the translating ribosome is sufficient to reactivate the backtracked EC by physically pushing it forward. This is likely to have important implications for synchronizing the rates of coupled transcription and translation (3), as well as minimizing the interference of backtracked ECs with replication forks (4), which may be especially important on genes with weak translation initiation signals and/or with rare codons, where the EC may fall into arrest before the leading ribosome catches up with it. Critically, our results show that stably backtracked ECs do not inhibit translation on their nascent mRNA, which otherwise would have drastic consequences for gene expression.

The contact distance between the coupled EC and ribosome measured by us in vitro is shorter than that proposed earlier based on a low-resolution cryo-EM structure of an “expressome”, a complex between a stalled EC and a coupled ribosome (14). Furthermore, the peptidyl-tRNA of the ribosome in the “expressome” was observed in the ribosome’s P-site, although interaction of the ribosome with the stalled EC on mRNA inhibits translocation of peptidyl-tRNA into the P-site (Fig. 2*B*), suggesting that in fact the ribosome of the “expressome” did not reach the stalled EC along the mRNA. Therefore, our results suggest that the interactions between the ribosome and the EC in the structure of the “expressome” are different from those occurring on contact between them on mRNA.

**Fig. 3.**
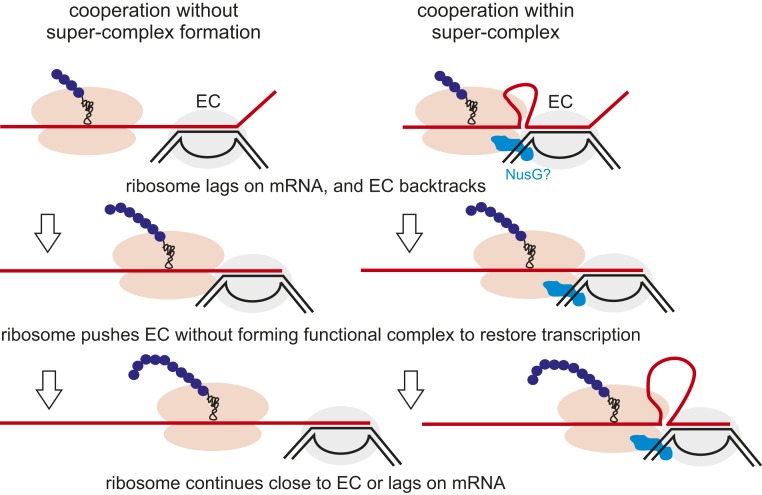
Models of transcription/translation coupling. The cooperation model implies contact between the ribosome and RNAP during pushing but no formation of a stable functional complex. However, cooperation may take place with or without formation of a structural supercomplex between the ribosome and EC.

Our results favor a “cooperation” model (Fig. 3) in which both the leading ribosome and the EC do not form a stable functional complex (such as the proposed rigid-state “expressome”) but rather move along the respective templates independently. In the event that the ribosome lags on the mRNA behind the EC and the EC falls into the arrest, the ribosome catches up with it and reactivates it. Our results also suggest that the presence of NusG and NusA do not affect the pushing ability of ribosome or the contact distance between the ribosome and the EC. However, we cannot exclude the possibility that the coupled ribosome and EC may form a structural supercomplex (possibly similar to the “expressome”) at a noncontact distance on the mRNA (i.e., mRNA looping between them), while still being able to move independently on their respective templates (Fig. 3). The formation of such a supercomplex would likely require NusG, which interacts with both RNAP and the ribosome and has been proposed to participate in coupling (10). In this case, however, this supercomplex would be distinct from the “expressome,” because there is no available space for NusG binding in the structure of the latter. We are currently investigating these possibilities.

## Materials and Methods

### Components of Coupled System.

Core RNAP was purified as described by Murakami (15), except the final gel filtration was omitted. Ribosomes, S100 (as a source of aminoacyl tRNA synthetases), EF-G, EF-Tu, EF-Ts, IF-1, IF-2, IF-3, formyl methionine transferase, and Met-RS were all purified as described previously (16–18). RelE was purified as described previously (9), using the plasmids kindly provided by Kenn Gerdes, Copenhagen University. The *greA* gene was cloned into the pET21 vector, expressed, and His-tagged GreA-purified as described previously (19). The *greB* gene was cloned into the pET28a vector and His-tagged GreB-purified as described previously (20).

Aminoacylation of tRNAs, formylation of Met-tRNA^Met^, and formation of ternary complexes (TCs; complexes of aminoacylated tRNA/EF-Tu/GTP) were performed as described previously (16–18).

Primers and the single-stranded DNA template (IDT) used to make a double-stranded template for mRNA synthesis by T7 RNAP are shown in *SI Appendix*, Fig. S1*B*. mRNAs (*SI Appendix*, Fig. S1*A*) were synthesized and purified as described previously (16–18). mRNA was either kinated with T4 kinase as described previously (16–18) or radiolabeled during transcription as described below. All DNA oligonucleotides for EC assembly (*SI Appendix*, Fig. S1*A*) were obtained from IDT, and NTPs were obtained from GE Healthcare.

### Coupling and Transcription and Translation in Coupled Systems.

Coupling of transcription and translation in vitro was carried out using the translation first coupled transcription–translation method developed by us (16–18) with some modifications. For a typical experiment (<10 reactions), translation was initiated using 16 pmol mRNA, 20 pmol ribosomes, 50 pmol fMet-tRNA^fMet^, 200 pmol IF-1, 200 pmol IF-2, 200 pmol IF-3, and 4 mM GTP in 50 μL of coupling buffer [CB; 10 mM Tris⋅HCl pH 7.4, 60 mM NH_4_Cl, 10 mM Mg(OAc)_2_, 6 mM β-mercaptoethanol] for 10 min at 37 °C. For the experiment shown in Fig. 2 *B* and *C*, initiation complex (M complex) was elongated by one codon to form an MF complex by the addition of 50 pmol Phe-tRNA^Phe^ TC, 200 pmol EF-G, and 4 mM GTP for 4 min at 37 °C. M or MF complexes were purified by centrifugation through a sucrose cushion as described previously (16–18). After centrifugation, the pellet was resuspended in 15 μL of CB, and 12 pmol template DNA and 16 pmol RNAP were added. After 15 min at 37 °C, 150 pmol nontemplate DNA preimmobilized (via biotin tag on 3′ end) on 5 µL of streptavidin beads slurry (Sigma-Aldrich) and equilibrated with CB were added, and EC formation and immobilization were completed in 15 min.

For the experiment shown in Fig. 1, transcription elongation of 5′ radiolabeled mRNA was performed for 10 min in the presence of 100 μM NTPs that brought that EC to the stalling and backtracking on encountering the streptavidin bead at the 3′ end of the nontemplate strand (Fig. 1*B* and *SI Appendix*, Fig. S1*A*). For the experiments shown in Fig. 2, 100 µM GTP, CTP, and 3′-dATP (Sigma-Aldrich) and 6 mCi α-[^32^P]-UTP (Hartmann Analytic; 3,000 Ci/mmol), were added for 10 min to radiolabel the mRNA and walk the EC to the abasic site of the template shown in Fig. 2*A* and *SI Appendix*, Fig. S1*A*. After transcription elongation, the coupled complexes were washed four times with 1 mL of CB. The volume of this master mix was adjusted to allow separation into a number (typically ∼10) of 10-μL reactions. For the experiment shown in *SI Appendix*, Fig. S3*A*, reactions were supplied with 50 μM inorganic pyrophosphate (Sigma-Aldrich) or with 1 μM GreA or 1 μM GreB for the indicated times at 37 °C.

Where indicated, after transcription, translation toward the EC was performed by the addition of 50 pmol of each corresponding TC, 200 pmol EF-G, and 4 mM GTP for 4 min. When indicated, 150 pmol NusG and 40 pmol NusA were added before translation. Then all coupled reactions (including those in which translation was not performed) were washed three times with 1 mL of CB. When indicated, 5 pmol GreB was added for 5 s, and reactions were washed again. Also where indicated, 20 pmol RelE was added for 10 min at 37 °C. For the experiment shown in *SI Appendix*, Fig. S4, the reactions corresponding to lanes 3 and 7 of Fig. 1*C* were supplied with 100 μM NTPs in the presence of 5 pmol GreB for the indicated times. All reactions were stopped by addition of an equal volume (10 μL) of formamide-containing stop buffer. Products of the reactions were separated by denaturing (8 M urea) electrophoresis in 10% polyacrylamide gel and analyzed by phosphorimaging and the use of ImageQuant software (GE Healthcare). All experiments were repeated at least three times.

### Quantification in Fig. 2*C*.

The efficiency of RelE cleavage at stop codons on different templates is the same and depends only on the availability of a UAG stop codon in the A-site of the ribosome. However, the observed cleavage efficiency also depends on the efficiency of coupling of transcription and translation complexes on each mRNA. Therefore, RelE cleavage before (at the V codon in the MF complex) and after (at the UAG stop codon) translation were quantified as a percentage of the total signal in the corresponding lane, and RelE cleavage at the stop codons was normalized between different mRNAs by dividing them by the efficiencies of RelE cleavage at the V codon in MF complexes on corresponding mRNAs. The data plotted are means, and error bars represent SDs from 3 to 13 experiments.

### Data Availability.

All data are presented in the main text and *SI Appendix*. The plasmids and strains constructed in this study are available on request.

## Supplementary Material

Supplementary File
