# Supplementary material for: Ribosome reactivates transcription by physically pushing RNA polymerase out of transcription arrest
Source: Proc Natl Acad Sci U S A. 2020 Apr 1;117(15):8462–7. doi: 10.1073/pnas.1919985117 (PMC7165469; doi:10.1073/pnas.1919985117)
Supplement: Supplementary File [file pnas.1919985117.sapp.pdf]

Supporting Information

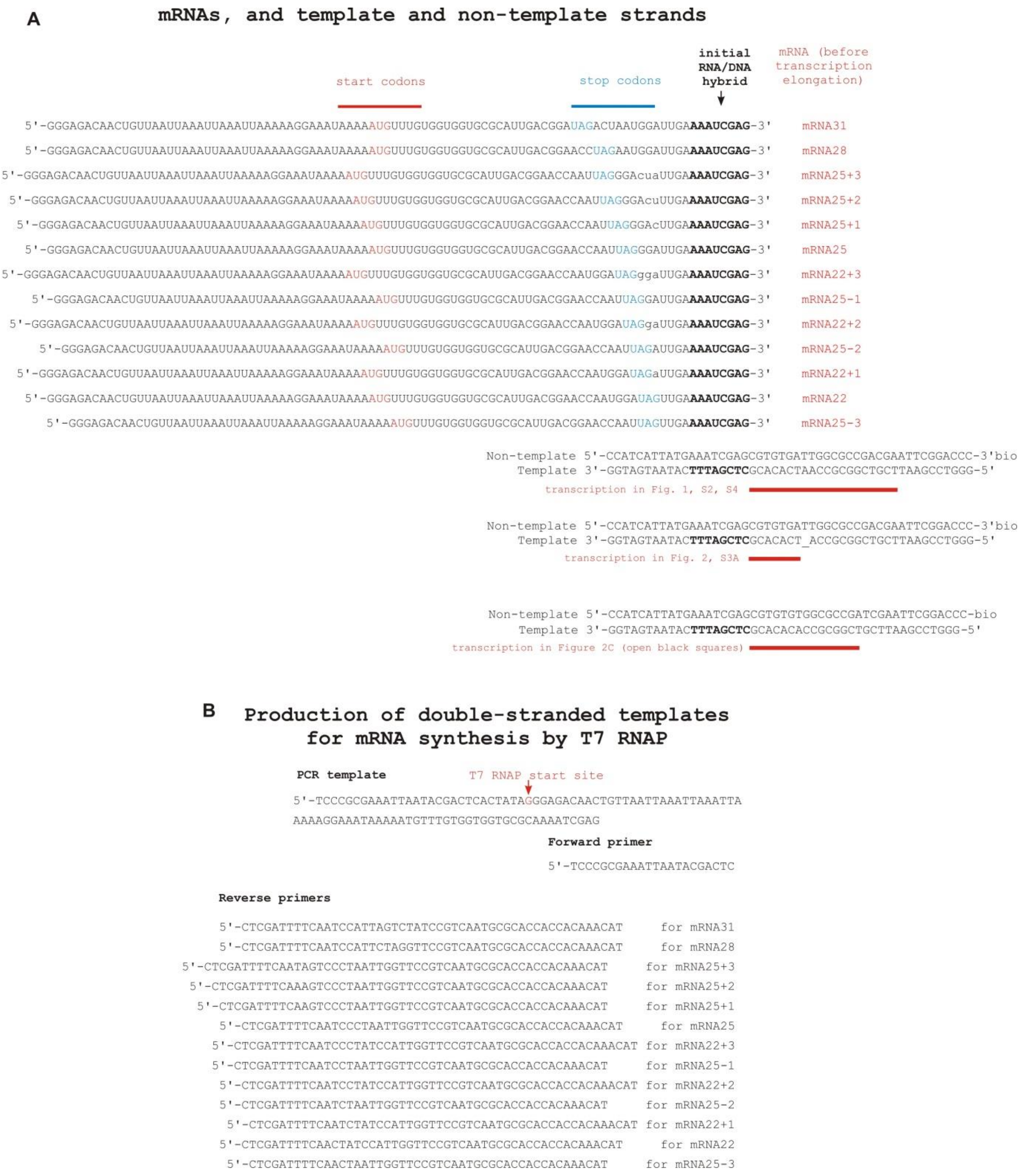

Fig. S1. Oligonucleotides used in the study. A. mRNAs, templates and non-templates used to assemble coupled system. **B**. Primers and the single-stranded template used to make double stranded templates for T7 transcription.

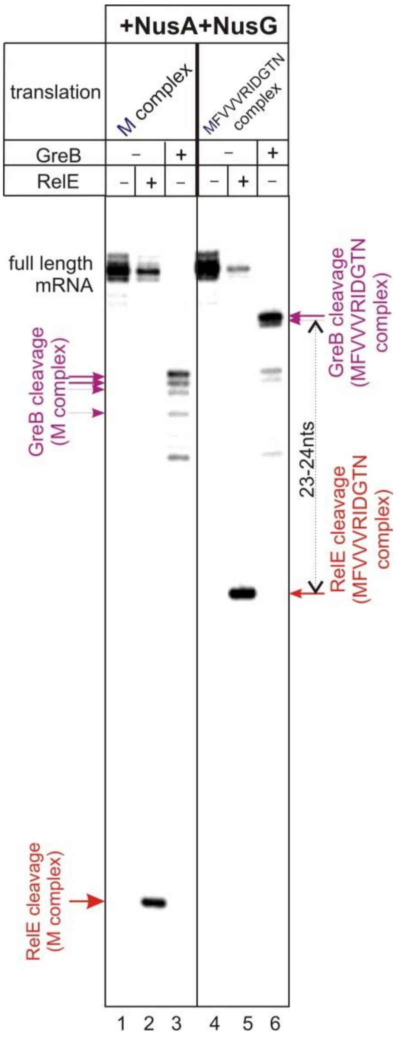

Fig. S2. NusA and NusG do not affect the rescue of the backtracked EC by the translating ribosome, and do not change the distance between the ribosome and RNAP active centers upon their contact.

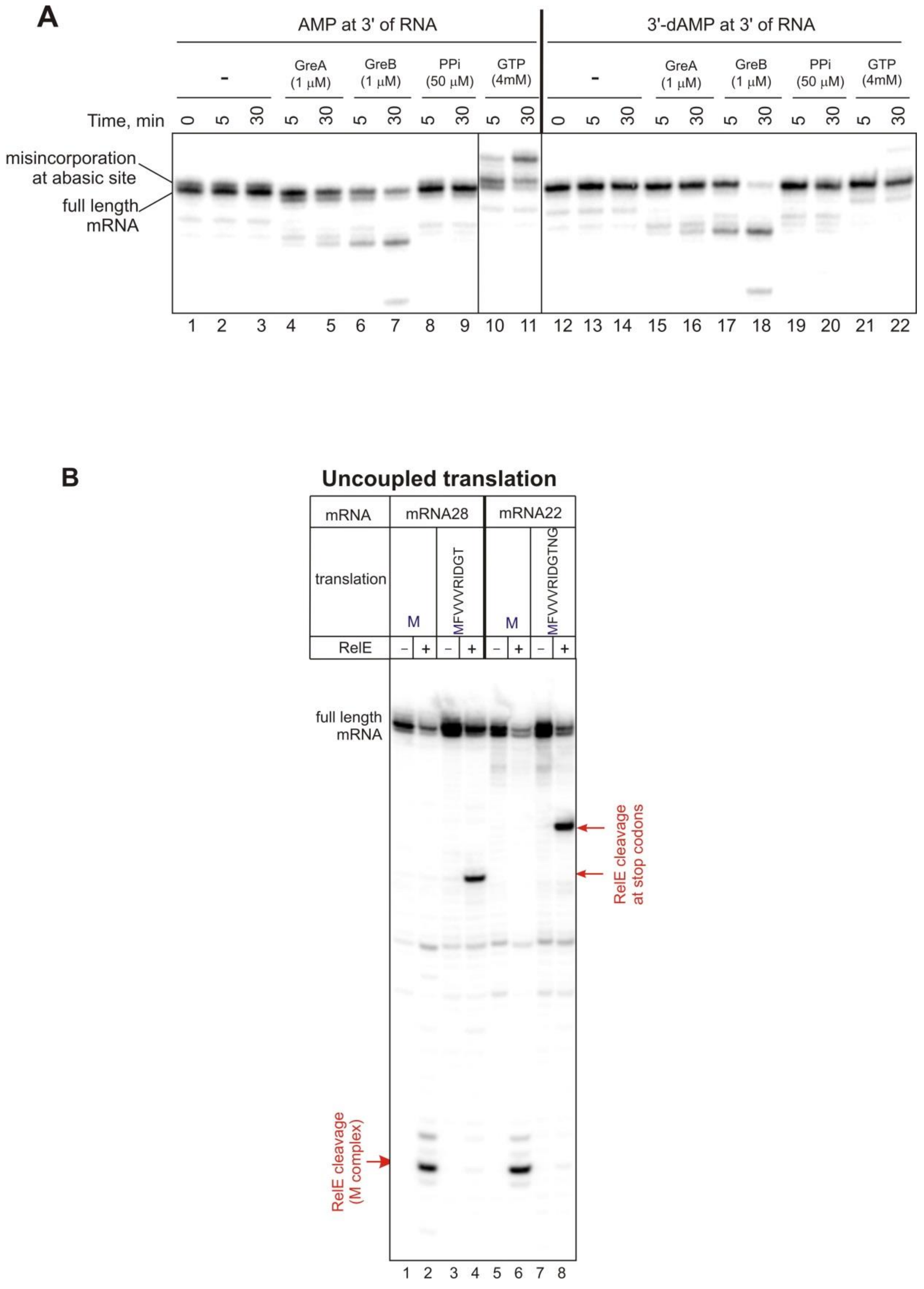

Fig. S3. Characterization of experimental system for distance measurements. A. Analysis of translocation state of the EC, by intrinsic, GreA and GreB cleavages (which take place only in backtracked states), pyrophosphorolysis (which takes place only in pre-translocated state), and GMP incorporation. Note that the fast cleavage by Gre factors happens only for RNA that is one nucleotide longer than full-sized mRNA (that incorporated NMP against abasic site). **B**. In uncoupled translation (without EC), RelE cleavage is not compromised irrespective of the proximity of ribosome to the mRNA’s 3’ end.

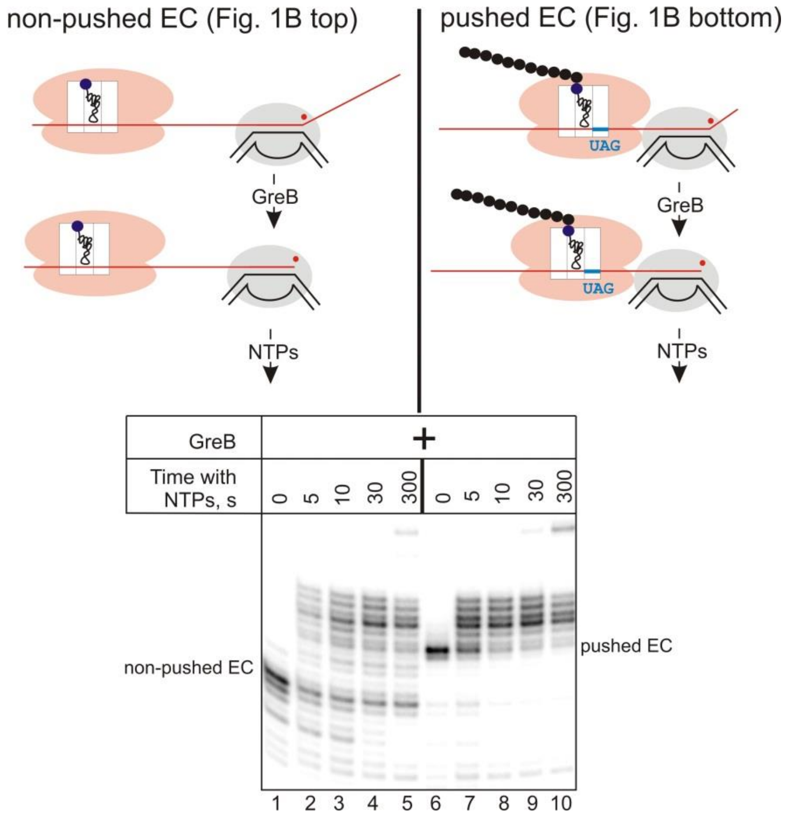

Fig. S4. Physical contact between ribosome and EC does not affect transcription elongation. C. Elongation in ECs contacting (right) or not-contacting (left) the ribosome on mRNA (see Fig. 1B for detailed scheme). Transcription was performed in the presence of GreB to reactivate the backtracked complexes.
